# Physicochemical properties and micro-interaction between micro-nanoparticles and anterior corneal multilayer biological interface film for improving drug delivery efficacy: the transformation of tear film turnover mode

**DOI:** 10.1080/10717544.2023.2184312

**Published:** 2023-03-03

**Authors:** Huamei Li, Fuda Dai, Hanyu Liu, Qi Tao, Jie Hu, Yangrong Zhang, Zhenping Xiao, Ilva D. Rupenthal, Huihui Li, Fan Yang, Wei Li, Huaqing Lin, Dongzhi Hou

**Affiliations:** a Guangdong Provincial Key Laboratory of Advanced Drug Delivery Systems and Guangdong Provincial Engineering Center of Topical Precise Drug Delivery System, College of Pharmacy, Guangdong Pharmaceutical University, Guangzhou, Guangdong, China; b CAS Key Laboratory of Mineralogy and Metallogeny & Guangdong Provincial Key Laboratory of Mineral Physics and Materials, Guangzhou Institute of Geochemistry, Chinese Academy of Sciences (CAS), Guangzhou, P.R. China; c Buchanan Ocular Therapeutics Unit, Department of Ophthalmology, New Zealand National Eye Centre, Faculty of Medical and Health Sciences, University of Auckland, Auckland, New Zealand; d Guangzhou Institute for Drug Control, Guangzhou, P.R. China

**Keywords:** Glaucoma therapy, tear film turnover, micro-interaction, montmorillonite, micro-nanoparticles

## Abstract

Recently, various novel drug delivery systems have been developed to overcome ocular barriers in order to improve drug efficacy. We have previously reported that montmorillonite (MT) microspheres (MPs) and solid lipid nanoparticles (SLNs) loaded with the anti-glaucoma drug betaxolol hydrochloride (BHC) exhibited sustained drug release and thus intraocular pressure (IOP) lowering effects. Here, we investigated the effect of physicochemical particle parameters on the micro-interactions with tear film mucins and corneal epithelial cells. Results showed that the MT-BHC SLNs and MT-BHC MPs eye drops significantly prolonged the precorneal retention time due to their higher viscosity and lower surface tension and contact angle compared with the BHC solution, with MT-BHC MPs exhibiting the longest retention due to their stronger hydrophobic surface. The cumulative release of MT-BHC SLNs and MT-BHC MPs was up to 87.78% and 80.43% after 12 h, respectively. Tear elimination pharmacokinetics study further confirmed that the prolonged precorneal retention time of the formulations was due to the micro-interaction between the positively charged formulations and the negatively charged tear film mucins. Moreover, the area under the IOP reduction curve (AUC) of MT-BHC SLNs and MT-BHC MPs was 1.4 and 2.5 times that of the BHC solution. Accordingly, the MT-BHC MPs also exhibit the most consistent and long-lasting IOP-lowering effect. Ocular irritation experiments showed no significant toxicity of either. Taken together, MT MPs may have the potential for more effective glaucoma treatment.

## Graphical Abstract

**Figure:**
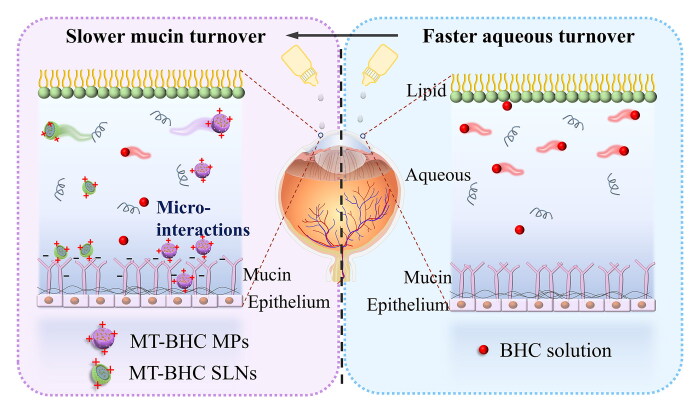

## Introduction

Glaucoma is the major cause of irreversible blindness worldwide due to damage to the optic nerve and the death of retinal ganglion cells (RGC) by intraocular hypertension (Tham et al., 2014; Sharif, 2021). Therefore, the current mainstay treatment aims to reduce intraocular pressure (IOP) through daily eye drops, laser treatment to the trabecular meshwork, or surgical operation (Quigley, 2011; Weinreb et al., 2014; Rahic et al., 2020). At present, the most commonly used clinical treatment involves eye drops containing prostaglandin analogs or β-adrenergic antagonists, either by increasing aqueous humor outflow or by reducing aqueous humor production (Quigley, 2011). However, daily eye drop administration results in poor patient compliance with the poor drug bioavailability of less than 5% also posing a problem (Schwartz GF & Quigley, 2008). The eye has several defense mechanisms in place, especially the corneal epithelium (50 µm thickness), which is made up of 5–6 layers of tightly connected cells. The flattened squamous superficial cells located in the outermost layer are the most important obstacle to drug delivery (Nowell & Radtke, 2017). However, drugs do not only need to overcome the static barrier of the cornea, but also the dynamic barrier of the tear film. The tear film consists of the outermost lipid, the middle aqueous, and the inner mucin layers. The aqueous layer accounts for 98% of the tear film and is renewed under blinking at a turnover rate of 16% min^−1^ (i.e. approximately 6 min for complete renewal), leading to rapid washout and thus low bioavailability of the vast majority of eye drops (Chang & Lee, 1987). At the same time, interactions of the drug with enzymes, mucins, and proteins in the aqueous layer of the tear film result in nonspecific binding, making it difficult for drugs to reach their target site (Van Haeringen, 1981; Sebbag et al., 2019). Ocular surface mucins are a negatively charged discontinuous reticular gel structure composed of mucins that adhere to microvilli and glycocalyx structures on the corneal and conjunctival surface extending into the tears (Rolando & Zierhut, 2001). Due to this adhesion, the turnover time of the mucin layer on the glycolytic structure is generally 6 to12 h (Roque et al., 2018), which is considerably longer than the turnover rate of the aqueous tear layer (about 6 min) (Chang & Lee, 1987). This allows drug molecules to remain on the ocular surface for a longer period when trapped in the mucin network structure, providing a good idea for the design of ocular mucosal drug delivery.

To improve patient compliance and drug bioavailability, novel drug delivery systems (NDDS) have been developed over the past decades to prolong the contact time between the drug and the ocular surface, slow drug elimination, and protect the drug from ocular enzymes (Hitoshi et al., 1996). At present, the most commonly used NDDS include liposomes, nanoparticles, microspheres (MPs), dendrimers, microneedles, and nanocrystals (Gote et al., 2019). Solid lipid nanoparticles (SLNs) have also shown great potential as sustained release and drug targeting systems (Schwarz et al., 1994). The main components of SLNs are solid lipids, which have the additional advantage of a longer residence time on the corneal surface (Janagam et al., 2017). Results from our previous studies have shown that SLNs have the potential to prolong the precorneal residence time of drugs compared to other polymeric nanoparticles (Li et al., 2018; Liu S et al., 2020). Moreover, polymer MPs have been shown to have low toxicity, drug stability, good sustained release performance, and excellent anterior corneal retention ability (Bravo-Osuna et al., 2016). Our earlier study observed an approximately 1.6-fold increase in the residence time of the polymer MPs on the ocular surface, with improved bioavailability compared to the commercial preparation Betoptic®S eye drops (Liu H et al., 2021). Therefore, these two particulate carriers were selected for the current study.

Montmorillonite (MT) is an inorganic clay mineral with a three-layer flake structure, with the upper and lower layers being a silicon-oxygen tetrahedron, and the middle layer being an aluminum octahedron, which facilitates ion exchange between the layers (Ertem et al., 2010; Baek et al., 2012; Sandri et al., 2014; Park et al., 2016). Compared with other types of clay minerals, MT has the largest cation exchange capacity, specific surface area, and initial layer spacing (Bera et al., 2019; Zhu et al., 2021). Its unique layered structure enables it to insert drug molecules through electrostatic interactions and control drugs release by ion exchange with other ions present in biological fluids (Radmanesh et al., 2019; Xu et al., 2020). In this study, the water-soluble drug betaxolol hydrochloride (BHC) was loaded into the interlayer of MT by ion-exchange with the complex (MT-BHC) further incorporated into MPs and SLNs.

So far, there have been many literature reports that improving the precorneal retention of a formulation significantly improves drug bioavailability, but few articles have investigated the mechanism of the retention. This research attempts to compare the effect of several physicochemical parameters (zeta potential, rheology, surface tension, contact angle, diffusion time, and hydrophobicity) on the micro-interactions with tear film mucins and corneal epithelium cells to reveal the mechanism of prolonged precorneal retention time. Tear elimination pharmacokinetics study was performed to investigate the effect of tear film component turnover on the drug behavior in vivo. In addition, a glaucoma rabbit model was used to evaluate the IOP-lowering efficacy of MT-BHC SLNs and MT-BHC MPs in comparison to a BHC solution. Finally, ocular biocompatibility of the formulations was investigated. Overall, these studies may provide some suggestions for the selection and clinical design of ocular mucosal drug delivery systems.

## Materials and methods

### Materials

MT was purchased from Zhejiang Sanding Co., Ltd. (Shaoxing, China) and BHC (GC > 99%) was purchased from Haohua Industrial (Shandong, China). Montmorillonite complexes loaded with betaxolol hydrochloride (MT-BHC) were obtained by ion exchange of betaxolol hydrochloride in montmorillonite. Eudragit (EUD) RS PO and Eudragit RL PO were purchased from Degussa (Essen, Germany). Glycerin monostearate (GMS) was purchased from Aladdin Industrial Corporation. Soybean phospholipid (SPL) was obtained from AVT Pharmaceutical Co., Ltd. (Shanghai, China). Rose Bengal was purchased from Macklin (Shanghai, China). All other chemical reagents used in the study were of HPLC or analytical grade.

New Zealand white rabbits of either sex, weighing 1.5–2.0 kg, were purchased from the Laboratory Animal Center of Southern Medical University (Guangzhou, China), and all animal experiments were conducted in accordance with the Institutional Animal Care and Use Committee of Guangdong Pharmaceutical University (approval number of the animal experiment protocols is gdpulac2020122).

### Manufacture of MT-BHC MPs and MT-BHC-SLNs

Based on our previous studies (Tian et al., 2018), MT-BHC MPs were prepared by the emulsion–solvent evaporation method. Briefly, 200 mg of EUD RS PO, 200 mg of EUD RL PO, 50 mg of BHC, and 30 mg of MT-BHC were completely dissolved in an acetonitrile solution containing 80 mg of triethyl citrate, 80 mg of glycerol, and 124 mg of Tween 80 (O_1_ phase). The O_1_ phase was emulsified by slowly dropping into the O_2_ phase consisting of 248 mg of spirulina 80 and 10 mL of liquid paraffin and mixed rapidly with a stirrer (XW-80A, Haimen Kylin-Bell Lab Instruments Co., Ltd.), then sonicated (JY 92-II, Ningbo Scientz Biotechnology Co., Ltd. China) for 15 min in an ice bath with a ultrasonic power of 53% at 5-s intervals and stirred at 800 rpm for 1 h in the ice bath and later at RT for 0.5 h, followed by adjusting the speed to 650 rpm until a clear mixture was obtained. The resulting MPs were washed with *n*-hexane to remove any excess oil content on the surface, recovered by centrifugal filtration, and air-dried at room temperature. The MT-BHC MPs eye drops were prepared by dispersing the MPs powder in a sarcosine phosphate buffer containing a co-suspension agent and adding 3.1% mannitol (mass/vol) and 0.1% (mass/vol) Tween 80 to adjust the osmotic pressure and viscosity.

**Figure 1.**
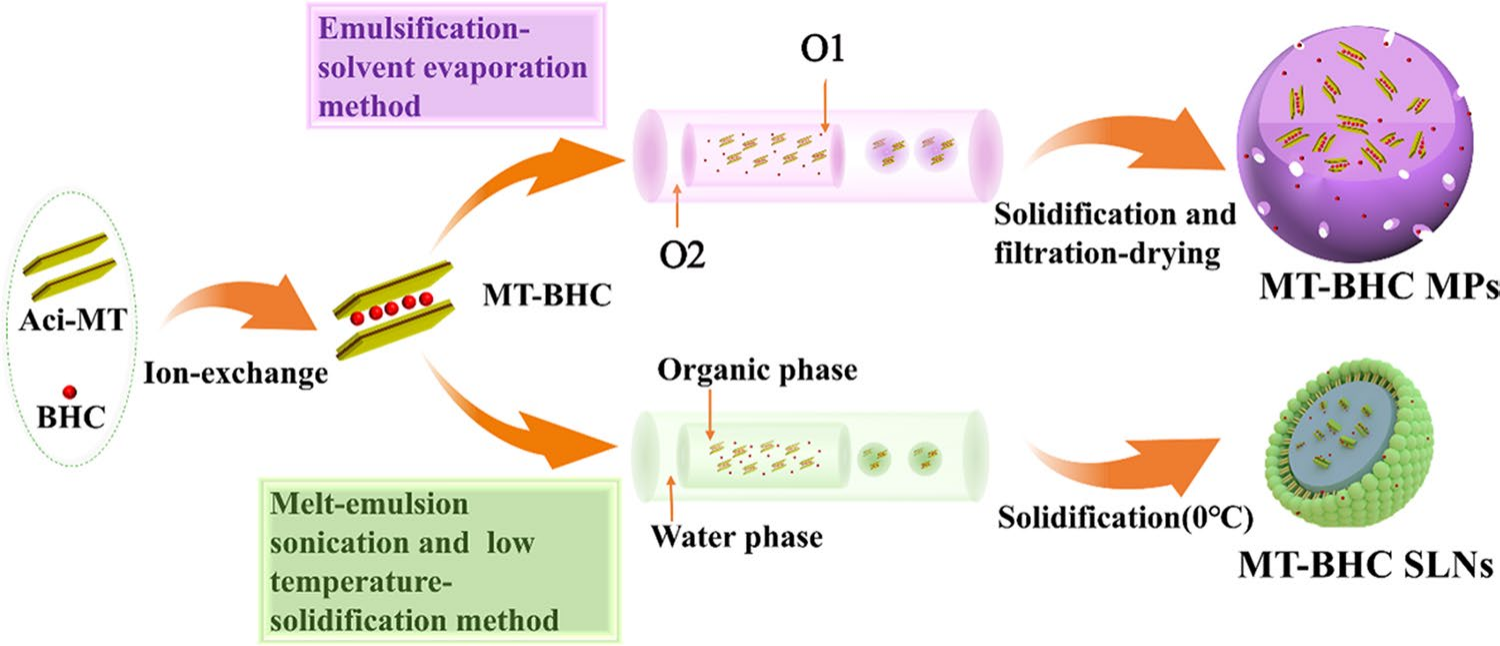
Schematic diagram of the preparation processes of MT-BHC MPs and MT-BHC SLNs.

MT-BHC SLNs were prepared by the melt-emulsion sonication and low temperature-solidification method in line with our previous research (Liu S et al., 2020; Han et al., 2021). Briefly, the organic phase was composed of 30 mg of BHC, 30 mg of GMS, and 100 mg of SPL dissolved in 5 mL of ethanol under heating at 75 °C. The water phase was a mixture of 200 mg of Tween 80, 100 mg of PEG-400, and 5 mg of sodium deoxycholate. First, 5 mg of MT-BHC complex was added to the organic phase and then ice bath ultrasonication for 10 min, injected into the aqueous phase at 75 °C under stirring at 800 rpm. Next, the stirring speed was increased to 1000 rpm and the mixture was stirred until an initial emulsion was formed. To maintain the stable morphology of SLNs, the initial emulsion was quickly injected into a 3.6% (wt/vol) aqueous mannitol solution at 0 °C and stirred for 2 h. The obtained SLNs were stored in a refrigerator at 4 °C. ***Figure 1*** shows a schematic of the two preparation methods.

### Characterization

The osmolarity of MT-BHC MPs and MT-BHC SLNs was tested by an osmometer (Osmomat Basic 3000, GonotecGmBTH, Germany), and the pH was measured using a pH meter (PHS-3C, INESA Scientific Instrument Co, Ltd.). Results were presented as an average value of the data obtained by triplicate measurements under the same conditions. Morphological characterization of MT-BHC MPs and MT-BHC SLNs was performed by scanning electron microscopy (SEM, Sigma 300, Carl Zeiss, Germany). Before testing, samples were diluted with deionized water to a suitable volume, then dropped on the silicon wafers of the sample table, placed in a vacuum drying oven at a drying temperature of 25 °C and gold spraying.

#### Characterization of MT-BHC MPs

Mean particle size, PDI, and zeta potential (ZP) were tested using a laser diffraction particle size detector (Mastersizer, Malvern, UK). The MT-BHC MP powder was demulsified with a dichloromethane–methanol solution (dichloromethane:methanol 1:9), and the absorbance of the sample was determined at 273 nm by UV spectrophotometry (UV-1800, Shanghai Mapada Instruments Co., Ltd., China) after filtration through a 0.22-µm filter. Entrapment efficiency (EE%) and drug loading (DL%) were calculated as follows:

$$\mathrm{EE}(\%)=\frac{W_{d}}{W_{t}\;}\times 100$$

$$\mathrm{DL}(\%)=\frac{W_{d}}{W_{s}}\times 100$$

where *W*_d_ is the total drug content, *W*_t_ is theoretical drug content, and *W*_s_ is the weight of MT-BHC MPs.

#### Characterization of MT-BHC SLNs

Mean particle size, PDI, and ZP were determined using a laser diffraction particle size detector (Delsa™ Nano C, Beckman Coulter, Inc. USA) with samples diluted in MilliQ water. Entrapment efficiency (EE%) and drug loading (DL%) were measured by the dialysis method using a dialysis bag (MWCO 8,000 to 14,000 Da). The absorbance at 273 nm was measured after filtration and calculations were performed as follows:

$$\mathrm{EE}(\%)=\frac{W_{d}}{W_{t}\;}\times 100$$

$$\mathrm{DL}(\%)=\frac{W_{d}}{W_{s}}\times 100$$

where *W*_d_ is the content of drug encapsulated, *W*_t_ is theoretical drug content, and *W*_s_ is the weight of MT-BHC SLNs.

#### In vitro release study

The in vitro BHC release from MPs and SLNs was measured by the dialysis method (Onyebuchi & Kavaz, 2019). A dialysis bag (MWCO 8000–14,000 Da) containing 4 mL of sample (BHC content of 2.8 mg/mL) was fastened at both ends and placed in 100 mL of simulated tear fluid (STF) composed of 0.678 g NaCl, 0.138 g KCl, 0.218 g NaHCO_3_, and 0.008 g CaCl_2_ 2H_2_O (Khan et al., 2018). The entire system was placed in an air bath at 34 °C and 120 rpm, and sealed with cling film to prevent evaporation of the dialysis medium. A volume of 5 mL of the release medium was removed, filtered through a 0.22-μm filter at predetermined time points (0.25, 0.5, 0.75, 1, 1.5, 2, 2.5, 3, 4, 5, 6, 7, 8, 9, 10, 11, and 12 h), and replaced with 5 mL of fresh STF. Finally, the amount of drug released was determined by measuring the absorbance at 273 nm using UV spectrophotometer. Release studies were performed in parallel with samples collected in triplicate.

### In vivo fluorescence tracing study

Ocular retention time was assessed by tracking changes in fluorescence intensity on rabbit eyes (Liu S et al., 2016). Formulations were prepared using 0.2% sodium fluorescein instead of BHC. Twelve healthy New Zealand white rabbits without eye disease were randomly divided into three groups (BHC solution, MT-BHC SLNs, and MT-BHC MPs). After application of 100 μL of formulations containing fluorescein sodium, rabbit eyes were manually closed for 10 s. The change in fluorescence intensity on the corneal surface was observed using a slit lamp under cobalt blue light (YZ5S, Liuliu Vision Technology Co., Ltd.).

### Physicochemical properties

#### Rheology

The rheological behavior of MT-BHC SLNs and MT-BHC MPs suspension was measured at 34 °C at a shear rate of 0–200 s^−1^ using a rotational rheometer (Physica MCR301, Austria) with a smooth parallel plate system (Horvat et al., 2015). Furthermore, the dynamic viscoelasticity was measured at a frequency of 1–10 Hz. The Ostwald-de Wale power law equation was used to fit the rheological curves according to the following equation:

$$\tau =K\;{\dot{\gamma }}^{n}$$

where *K* is the viscosity coefficient of the fluid and *n* is the non-Newtonian index of the fluid.

#### Surface tension and contact angle

After euthanasia of healthy New Zealand white rabbits without eye disease, the eyeballs were immediately peeled off and preserved in normal saline. The surface tension and contact angle of BHC solution, MT-BHC SLNs, and MT-BHC MPs on the corneal surface were measured by hanging drop method (Suri et al., 2021) using an optical contact angle measurement instrument (SDC-100, Dongguan Shengding Precision Instrument Co., Ltd, China). The preparation was slowly expelled from a 1-mL syringe with a needle of outer diameter 0.51 mm and the instantaneous state of the droplet leaving the needle was captured, recorded the surface tension analyzed on the display at this time. For contact angle measurements, the rabbit eyeballs were placed under the syringe and the eye surface was kept moist. Captured the instantaneous state of droplet contact with the eyeballs after dropping, and contact angle was analyzed by measuring the angle between the droplet and the eye surface with software. The spreading time of the preparations on the ocular surface in vitro was calculated as the time from just touching the eyeball to the contact angle no longer changing. All experiments were performed in triplicate.

#### Rose Bengal adsorption assay

The surface hydrophobicity of MT-BHC MPs and MT-BHC SLNs was measured using the Rose Bengal (RB) binding method (Doktorovova et al., 2012). Different volumes of BHC solution, MT-BHC SLNs, and MT-BHC MPs were taken, and double the volume of RB solution (30 μg mL^−1^) was added. After incubating for 2 h away from light at room temperature, the mixture was centrifuged at 15,000 rpm for 30 min. Afterward, the sample was filtered (0.22 μm) and the free RB in the supernatant was measured using UV spectrophotometry. The RB concentration was determined using the following formula:

$$\frac{r}{a}=KN-Kr$$

where *r* is the concentration of RB bound to the particles, *a* is the concentration of RB at equilibrium, *K* is the binding constant to evaluate the hydrophobicity of the particle surface, and *N* is the maximum binding amount of RB.

### Tear elimination pharmacokinetics study

Nine healthy New Zealand rabbits were randomly divided into three groups. Each rabbit was given normal saline as control in the left eye and 100 μL of preparation (BHC solution, MT-BHC SLNs, and MT-BHC MPs) in the right eye. At specific time points, a 3 × 8 cm filter paper strip was gently placed in the rabbit’s upper eyelid for 1 min, then weighed (Sartorius bsa122s – cw, Germany) to determine the amount of tears. Subsequently, the filter paper strips containing tears were dried using a nitrogen blowing instrument (MD200-1, Hangzhou Aosheng Instrument Co., Ltd, China), then placed in an eppendorf tube and 70 µL of methanol was added and vortexed for 5 min, sonicated for 20 min, and centrifuged at 15,000 rpm for 20 min. And 50 µL of supernatant was taken to determine the content of BHC in the tears by HPLC (Agilent Instrument 1200, USA) with a column (Inertsil ODS-C18, 4.6 × 150 mm). It was the chromatographic conditions that mobile phase constitution was acetonitrile and 0.2% triethylamine (vol:vol = 3:7, pH= 3), flowing rate was 1.0 mL min^−1^, the column temperature was room temperature, detection wavelength 273 nm, sample volume 20 μL. The standard curve was obtained as *Y* = 3495·*X* + 1876 (*r*^2^ = 0.9991), and the linearity of the method was good in the concentration range of 1.00∼300.00 μg mL^−1^.

### Pharmacodynamics

An intraocular hypertension model was established by injecting compound carbomer solution (including 0.3% carbomer and 0.025% dexamethasone, pH = 4.0) into both left and right anterior chamber of the rabbit eyes (Huang et al., 2016). Healthy New Zealand white rabbits without eye disease were anesthetized by injecting 10% chloral hydrate solution (2.5–3 mL kg^−1^) through the ear margin vein and adding 1–2 drops of procaine hydrochloride. A volume of 0.1 mL aqueous humor was drawn from the anterior chamber with a 1-mL syringe, and 0.1 mL compound carbomer solution was injected immediately. After compound carbomer gelation, 1–2 drops of tobramycin were added to the ocular surface to prevent infection. Rabbits whose intraocular pressure (IOP) was continuously above 22 mmHg for 7 days were selected for the study. Finally, nine New Zealand white rabbits with successful glaucoma molding in both eyes were randomly divided into three groups of three rabbits each. BHC solution, MT-BHC SLNs, or MT-BHC MPs (100 μL, with 0.28 mg BHC) was given to the right eye and saline to the left eye (control). The IOP was measured at specified time points after drop administration using a tonometer (Tonovet plus, iCare, Finland). The area of IOP reduction curve was analyzed (Hathout et al., 2019; Abdel Azim et al., 2022). All measurements were evaluated by the same operator under the same environmental conditions.

### Ocular biocompatibility

#### Draize test

Nine healthy New Zealand white rabbits without eye disease were randomly divided into three groups. A volume of 0.05 mL saline was dropped into the left eye (control) whereas 0.05 mL of BHC solution, MT-BHC SLNs, or MT-BHC MPs with equivalent BHC concentration was administered into the right eye. Rabbit eyelids were manually closed for 10 s after administration. Eye examination, recording, and scoring were performed at 48 and 72 h after a single administration, and before daily administration in the 7 days of multiple administration experiment. The irritation of each preparation was evaluated according to the previously published scoring scheme (Bengani et al., 2020).

#### Histopathology examination

After the multiple administration experiment, New Zealand white rabbits were euthanized by injecting air into the ear vein. The cornea and sclera were separated after enucleation of the rabbit eyeball. The separated tissues were fixed in 4% polyformaldehyde, embedded in paraffin, sectioned into 10 µm section, and stained with hematoxylin and eosin (HE). The prepared tissue sections were observed under a light microscope (BK6000, Chongqing Optec Instrument Co., Ltd., China).

#### Blink experiment

Saline, BHC solution, MT-BHC MPs, and MT-BHC SLNs (0.05 mL) were dropped into the conjunctival sac of healthy New Zealand rabbits without eye disease. The number of blinks in 1 min was recorded after the rabbit eyes were manually closed for 10 s.

### Statistical analysis

All statistical analyses were performed using Origin 2022 software. Comparisons with *p* values less than .05 were considered statistically significant and are marked with an asterisk or well symbol. All data are expressed as the mean ± standard deviation (SD).

## Results and discussion

### Characterization of MT-BHC MPs and MT-BHC-SLNs

**Figure 2.**
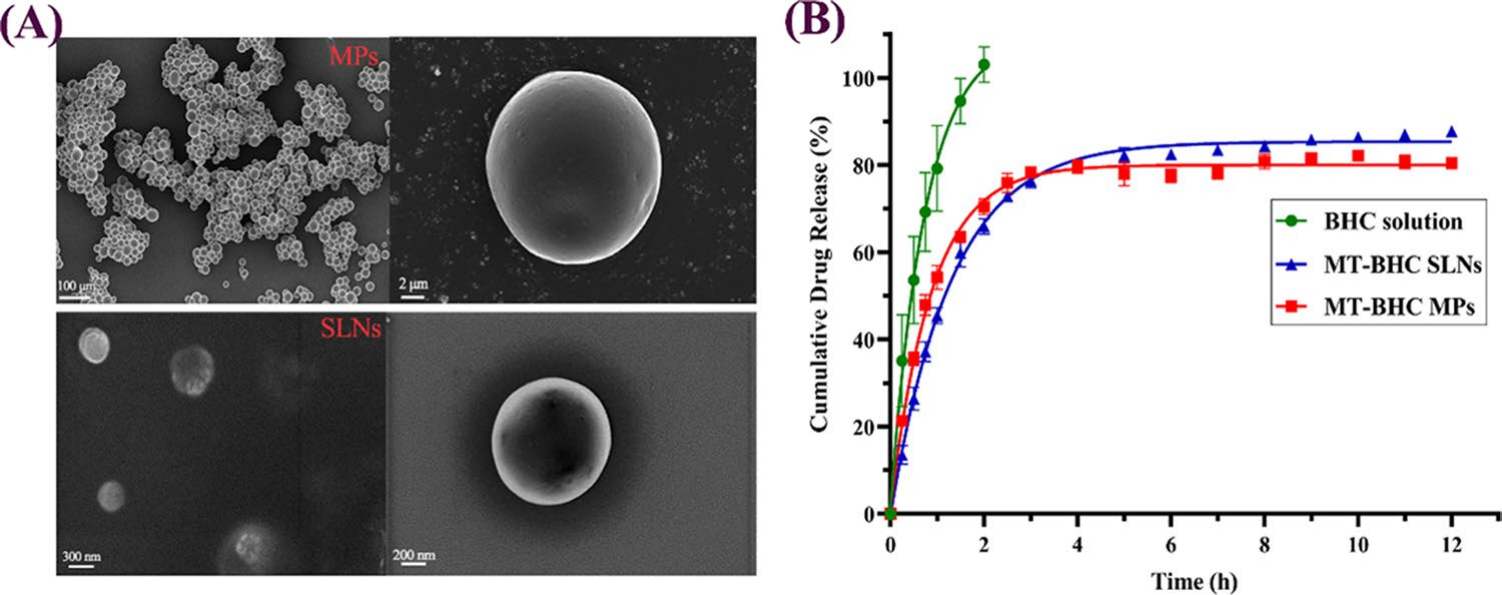
Characterization of MT-BHC MPs and MT-BHC SLNs. A: SEM images of MT-BHC MPs (top) and MT-BHC SLNs (bottom); B: Cumulative drug release from MT-BHC MPs, MT-BHC SLNs, and BHC solution (mean ± SD, *n* = 3).

Ophthalmic preparations must strictly meet defined requirements to avoid discomfort as well as drug loss associated with frequent blinking. ***Table 1*** illustrates that the adopted preparation method resulted in MT-BHC MPs with a mean particle size of around 10 μm, able to encapsulate BHC with an entrapment efficiency of around 84%. MT-BHC SLNs had an average particle size of around 547 nm with an entrapment efficiency of about 82%. The drug loading of MT-BHC MPs was 1.47 times that of MT-BHC SLNs. To maximize comfort, the pH value of the ophthalmic preparation should be the same as that of normal tears (about 7.4). Tears are almost isotonic with an osmotic pressure of around 300 mOsmol/kg, although eyes can withstand an osmotic pressure range of 248–370 mOsmol/kg (Kang et al., 2016). Hence, the pH and osmolarity of the above ophthalmic preparations met the requirements. Tear film and corneal epithelial cells are negatively charged, as such, the particle surface charge plays an important role. Both MT-BHC MPs and MT-BHC SLNs were positively charged, which not only prevented particle aggregation but also resulted in interactions with negatively charged mucins to enhance precorneal retention (Khames et al., 2019). As shown in ***Figure 2***(A), MT-BHC MPs and MT-BHC SLNs were uniform round-spherical particles with a little pothole on their surfaces under a scanning electron microscope. Although some aggregation of MT-BH MPs could be observed, no free drug crystals were found, indicating that the drug was loaded into the matrix.

**Table 1.** Physicochemical characteristics of MT-BHC MPs and MT-BHC SLNs (data expressed as mean ± SD, *n* = 3).

	MT-BHC MPs	MT-BHC SLNs
Particle size	9.82 ± 3.56 µm	547.70 ± 2.37 nm
ZP	8.58 ± 0.57 mV	7.84 ± 0.94 mV
EE%	85.43 ± 1.27	83.52 ± 0.91
DL%	6.23 ± 0.07	4.55 ± 0.05
pH	6.19 ± 0.16	6.07 ± 0.11
Osmolarity	300 ± 3 mOsmol/kg	301 ± 2 mOsmol/kg

### In vitro release study

The in vitro cumulative drug release from the BHC solution, MT-BHC MPs, and MT-BHC SLNs is shown in Figure 2(B). Drug from the BHC solution diffused rapidly out of the dialysis bag into the release medium with a cumulative release of 53.63% at 0.5 h, while the cumulative drug release from MT-BHC MPs and MT-BHC SLNs was only 35.55% and 24.41%, respectively. In the early phase, the drug from MT-BHC MPs was released slightly faster than from MT-BHC SLNs, most likely due to the dissociation of more drug molecules adsorbed on the microsphere surface. The cumulative drug release from MT-BHC MPs and MT-BHC SLNs after 12 h was 80.43% and 87.78%, respectively, suggesting that both could maintain longer-lasting drug release relative to the BHC solution.

### In vivo fluorescence tracing study

**Figure 3.**
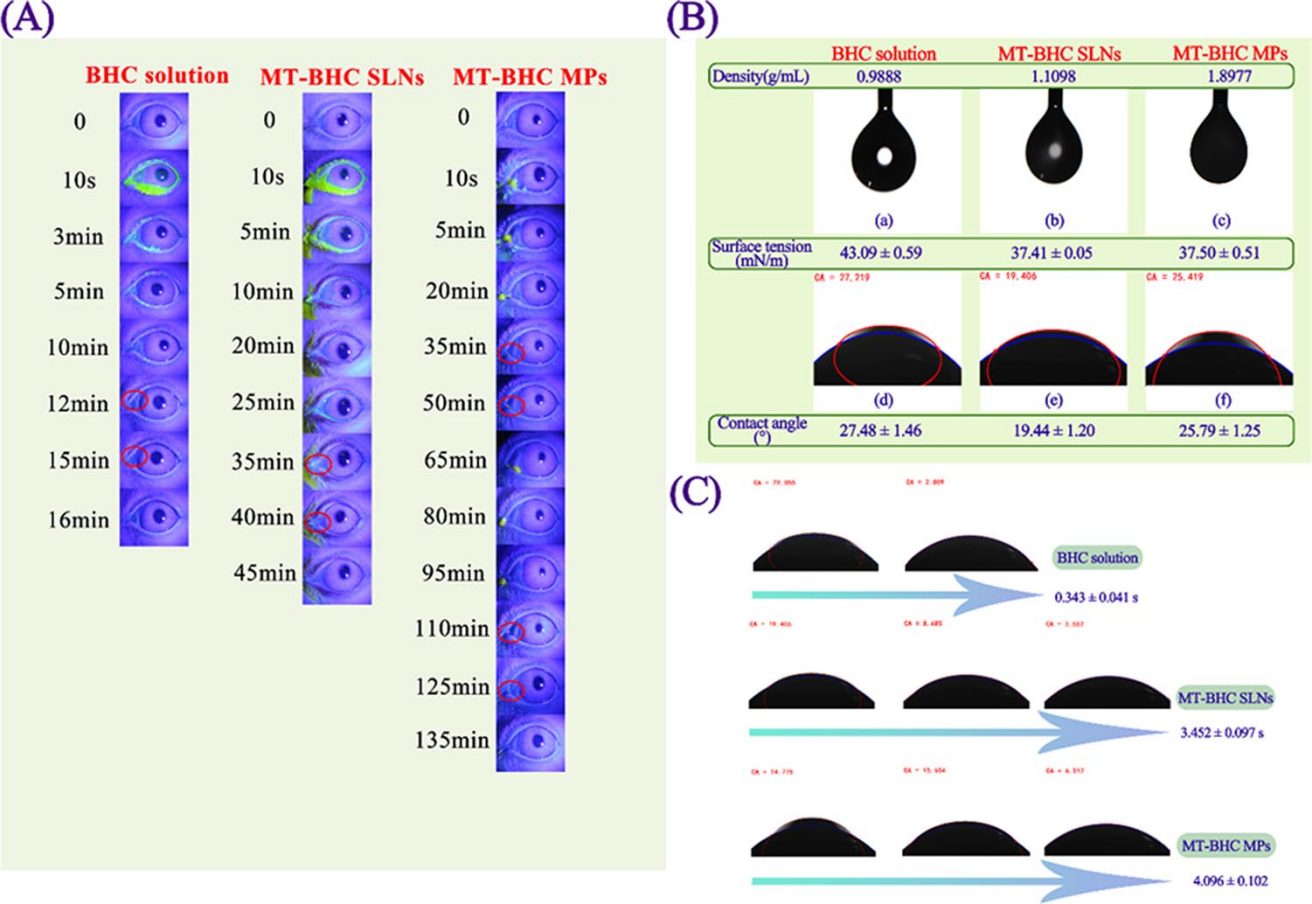
In vivo fluorescent tracing and physicochemical properties of BHC solution, MT-BHC SLNs, and MT-BHC MPs. A: Precorneal retention of the BHC solution, MT-BHC SLNs, and MT-BHC MPs eye drops using the fluorescence tracing method. No fluorescence was found after application of the BHC solution after 16 min, the MT-BHC SLNs after 45 min, and the MT-BHC MPs after 135 min. B: Surface tension and contact angle of the BHC solution, MT-BHC SLNs, and MT-BHC MPs eye drops. C: The spreading time of BHC solution, MT-BHC SLNs, and MT-BHC MPs eye drops on isolated rabbit cornea.

Sodium fluorescein was loaded into the preparations to observe its retention time on the ocular surface. As shown in ***Figure 3***(A), a high amount of fluorescence was observed on the ocular surface within 10 s after administration of the BHC solution. However, due to blinking and nasolacrimal drainage, the majority of the fluorescence disappeared from the ocular surface within 3 min but was found in the rabbit nose. After 16 min, no fluorescence was visible on the ocular surface in the BHC solution group. However, higher fluorescence was still observed after 20 min for the MT-BHC MPs and MT-BHC SLNs groups. No fluorescence signal was detected in the MT-BHC SLNs after 45 min, while a signal was still visible for the MT-BHC MPs group until 135 min. This suggests that the retention time of MT-BHC MPs and MT-BHC SLNs was significantly longer than that of the BHC solution. To explore the reasons why the particle preparations significantly prolong the precorneal retention, we first studied the following physicochemical properties of the three preparations.

### Physicochemical properties

#### Rheology

Rheological studies evaluated the viscosity of micro-nanoparticles and their fluid properties. ***Table 2*** lists the rheological behavior of MT-BHC MPs and MT-BHC SLNs. After fitting the power law equation, the *n* values of MT-BHC MPs and MT-BHC SLNs were both less than 1, suggesting the shear-thinning pseudoplastic fluid (Mahdi et al., 2011). Pseudoplastic behavior is one of the important properties of ocular formulations. The ocular shear force generated during blinking induces a tendency for the micro-nanoparticles to align in the direction of flow, reducing the resistance to flow and promoting the spreading of the formulation. However, after the disappearance of ocular shear force, the formulations gradually reorganizes and is maintained at the ocular surface (Wang et al., 2019). In other words, higher initial viscosity endows the MPs and SLNs with the ability to resist being washed away by tears. After that, the reduced viscosity of MPs and SLNs under blinking (4250–28,500 s^−1^) (Paradkar & Parmar, 2017), not only reduces discomfort and irritation, but might also promote the spreading on the ocular surface. Pseudoplastic MPs and SLNs formulations are better than Newtonian BHC solution to maintain higher drug concentrations on the ocular surface, thus predicting better efficacy.

**Table 2.** Rheological behavior of MT-BHC SLNs and MT-BHC MPs eye drops (mean values ± SD, *n* = 3).

Sample	Power law equations	non-Newtonian index (*n*)	*r* ^2^	Fluid type
MT-BHC MPs	$\tau =0.037{\dot{\gamma }}^{0.747}$	0.747	0.9998	pseudoplastic fluid
MT-BHC SLNs	$\tau =0.006{\dot{\gamma }}^{0.755}$	0.755	0.9900	pseudoplastic fluid

#### Surface tension and contact angle

Ophthalmic preparations with a lower surface tension indicate better spreading performance, and with a smaller contact angle are more likely to wet the lipid layer of the tear film and the surface of the hydrophobic corneal epithelium (Hotujac Grgurevic et al., 2017). The surface tension of the normal air/tear interface is 43.6 mN/m (Mullertz et al., 2021), and the surface tension of ophthalmic preparations is generally in the range of 34.4–70.9 mN/m (Biro et al., 2018). The surface tension of MT-BHC MPs and MT-BHC SLNs was 37.50 ± 0.51 mN/m and 37.41 ± 0.05 mN/m (Figure 3(B)), respectively, which met the requirements of ophthalmic preparations and was significantly less than that of the BHC solution (43.09 ± 0.59 mN/m). The lower surface tension of MT-BHC MPs and MT-BHC SLNs led to better spreading on the lipid layer and hydrophobic corneal epithelium, contributing to increased micro-interaction with tear film mucins and corneal epithelium, thus prolonging the precorneal retention time. The contact angles of BHC solution, MT-BHC MPs, and MT-BHC SLNs were far below 90°, which further confirmed that the three preparations had good wettability. Isolated corneal spreading results indicated that the in vitro corneal spreading time of MT-BHC MPs (4.096 ± 0.102 s) and MT-BHC SLNs (3.452 ± 0.097 s) was significantly (*p* < 0.05) longer than that of the BHC solution (0.343 ± 0.041 s), with MT-BHC MPs having the longest spreading time (Figure 3(C)), again providing more opportunities for micro-interactions between positively charged particles and negatively charged mucins in the tear film. The longer spreading time of MT-BHC MPs than MT-BHC SLNs also indicated that MPs were less fluid than SLNs, i.e., higher viscosity, which might contribute to their longer retention on the ocular surface. This is in agreement with the rheological results. To further investigate the better affinity between the MPs prepared with the polyacrylic resin as a carrier and the hydrophobic corneal epithelium compared to the SLNs prepared from lipids with similar properties to the biofilms, we designed Rose Bengal adsorption experiments.

#### Rose Bengal adsorption assay

As a hydrophobic dye, Rose Bengal combines with the hydrophobic groups on the surface of the particles. The higher the binding constant *K* value, the closer the binding, and the stronger the hydrophobicity of the particle surface. From ***Table 3***, it can be seen that the *K* value of MT-BHC MPs is higher than that of MT-BHC SLNs, indicating that the hydrophobicity on the surface of MT-BHC SLNs is lower than that of MT-BHC MPs (i.e. the surface of SLN is more hydrophilic). The lipid material in the prescription is glycerol monoacetate, a surfactant with a weak surface activity that contains a large number of hydrophilic groups, while the carrier material of MT-BHC MPs is a quaternary methacrylic resin containing lots of alkyls. In addition, it may also be related to the hydration properties of the surface of MT-BHC MPs with polyacrylic resin as a carrier and MT-BHC SLNs with glycerol monostearate as a carrier. To sum up, hydrophobic groups on the particle surface could interact better with the hydrophobic components in the mucus resulting in mucoadhesion. When the highly hydrophobic MT-BHC MPs passed through the mucin layer to the corneal epithelium, they could interact with the highly hydrophobic corneal epithelial cells to prolong precorneal retention.

**Table 3.** Scatchard-plot equation and Rose Bengal binding constant values (*K*) for MT-BHC MPs and MT-BHC SLNs.

Sample	Scatchard-plot equation	Binding constant (*K*)
MT-BHC MPs	*y* = –343.03*x* + 4.3809	343.03
MT-BHC SLNs	*y* = –84.849*x* + 1.645	84.849

Thus, a suitable viscosity, lower surface tension and contact angle, longer precorneal spreading time, and higher hydrophobicity of the surface of the carrier material combined to facilitate the prolonged residence time of the formulation at the ocular surface. To confirm whether this translated into improved efficacy, tear pharmacokinetic experiments and IOP reduction pharmacodynamic experiments were performed.

### Tear elimination pharmacokinetics

**Figure 4.**
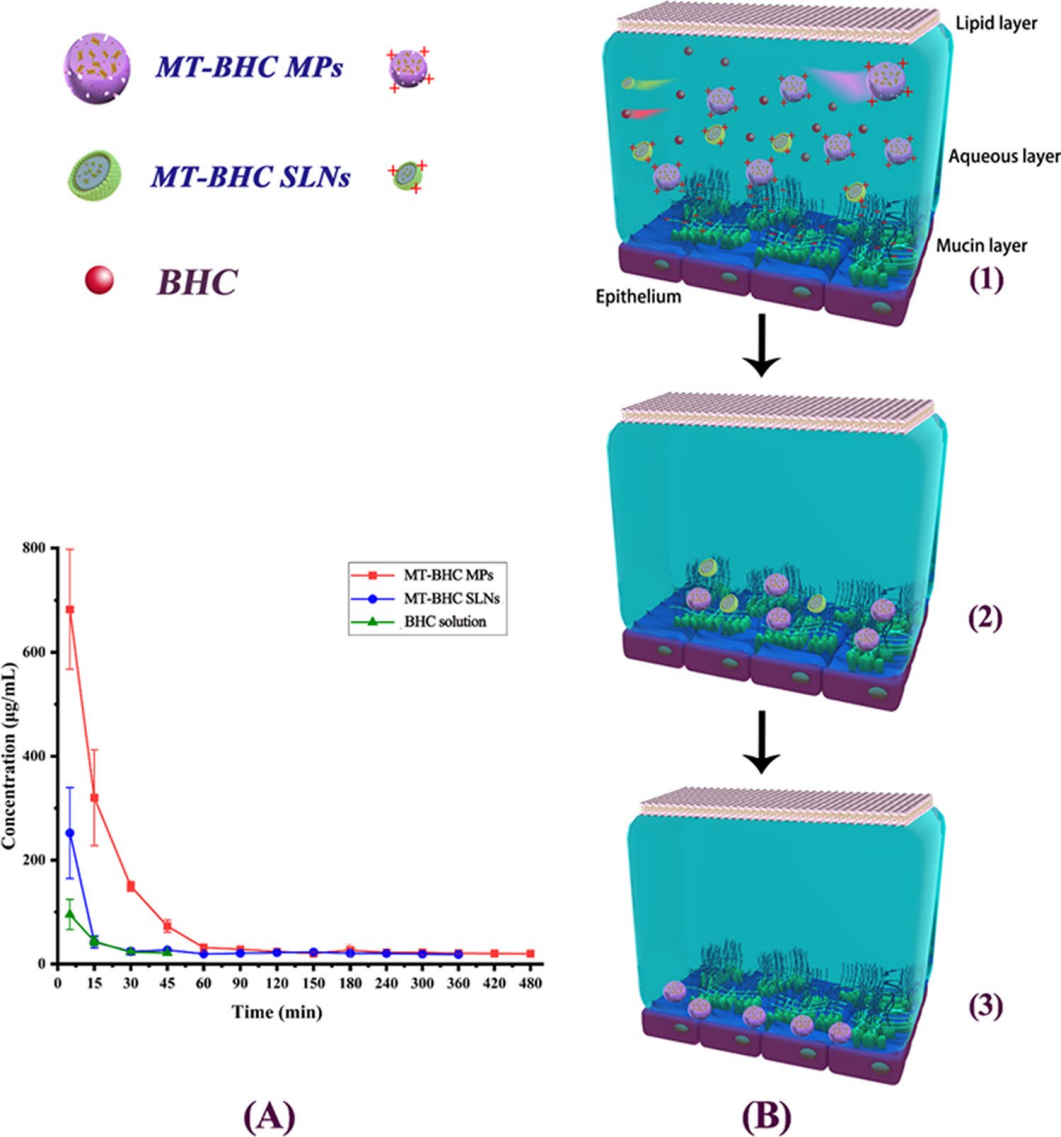
Tear elimination pharmacokinetics and schematic diagram of transformation for tear film turnover pattern based micro-interactions. A: Tear drug concentration-time curves after topical application of 100 μL BHC solution, MT-BHC MPs, and MT-BHC SLNs eye drops to rabbit eyes (mean ± SD, *n* = 3). B: Schematic representation of the proposed micro-interactions of the BHC solution, MT-BHC MPs, and MT-BHC SLNs with the ocular surface after topical administration. (1) In the early stage, some free drug molecules from the BHC solution, large MT-BHC MPs and small MT-BHC SLNs remain on the ocular surface. (2) Positively charged MT-BHC MPs, and MT-BHC SLNs interact with negatively charged tear film mucins suggesting that particles are no longer eliminated by aqueous flow. (3) After penetrating the mucin layer, MT-BHC MPs with higher hydrophobicity interacted with the corneal epithelium to maintain longer retention.

To further analyze the retention of particles on the ocular surface, we quantified the drug in the tears. ***Figure 4***(A) demonstrated the drug concentration–time curves after application of the BHC solution, MT-BHC MPs, and MT-BHC SLNs eye drops. The drug concentration after application of the BHC solution was only 92.89 μg/mL after 5 min, reducing to an amount below the detection limit at 45 min. The drug concentrations after application of MT-BHC SLNs and MT-BHC MPs were 252.13 μg/mL and 682.53 μg/mL after 5 min, respectively, which was much higher than that for the BHC solution (95.37 μg/mL). The drug could still be detected in the tears 360 min after MT-BHC SLNs and 480 min after MT-BHC MPs administration, which could be attributed to their better precorneal retention and the slow drug release over time.

The analysis of the area under the tear drug concentration–time curves (AUC) (***Table 4***) in tear fluid demonstrated significantly larger AUC for MT-BHC SLNs and MT-BHC MPs than the BHC solution, about 5.4 and 12.7 times that of the BHC solution, respectively. Significantly, AUC of MT-BHC MPs is 2.4 times larger than that of MT-BHC SLNs (*p* < 0.0001). This differences in the AUC indicates that both MT-BHC SLNs and MT-BHC MPs noticeably improved retention time on the ocular surface compared to the BHC solution.

**Table 4.** Date analysis of the tear elimination pharmacokinetics results.

	BHC solution	MT-BHC SLNs	MT-BHC MPs
Area under the Curve (AUC) ((μg·min/mL) ± SD)	1,762.0 ± 181.43	9,458.8 ± 536.21^****^	22,323 ± 1179.8^****####^

Mean values ± SD, *n* = 3 per group; ^*^ P< 0.05, ^**^ P< 0.01, ^***^ P< 0.001, and ^****^ P< 0.0001 vs. the BHC solution. ^#^ p< 0.05, ^##^ p< 0.01, ^###^ p< 0.001, and ^####^ P<0.0001 vs. the MT-BHC SLNs.

To further highlight the differences in drug elimination from the ocular surface, we propose the below mechanism of action (Figure 4(B)): The negatively charged mucin in the tear film forms a reticular structure with a certain mesh size and discontinuous parts, which allows certain sized particles to penetrate (Sigurdsson et al., 2013; Roque et al., 2018). The negatively charged polysaccharide part of the extracellular protein–polysaccharide complex of the corneal epithelium extends into the tear film, with the flow time of the glycocalyx structure being between 6 and12 h (Sigurdsson et al., 2013; Imperiale et al., 2018). The longer spreading time of the MT-BHC MPs gave them more time to interact with the negatively charged mucins. Moreover, the highly hydrophobic nature of the MT-BHC MPs enabled them to better penetrate the mucin layer and interact with the hydrophobic corneal epithelium, leading to better drug retention on the ocular surface for up to 8 h. In contrast, most of the drugs from the BHC solution were washed away by the aqueous layer of the tear film.

In fact, the precorneal elimination of BHC in solution was controlled by the renewal and flow of the tear film aqueous layer, which has a flow rate of 16% min^−1^, meaning that a complete renewal of the tears would be completed in approximately 6 min. Whereas positively charged MT-BHC MPs and appropriately sized MT-BHC SLNs are captured by the mucin layer, the slow physiological circulation of mucin lasts for several hours, thus altering the precorneal circulation pattern of the micro-nanoparticles and shifting drug clearance from being controlled by aqueous turnover to mucus turnover, achieving enhanced precorneal retention.

### Pharmacodynamics

**Figure 5.**
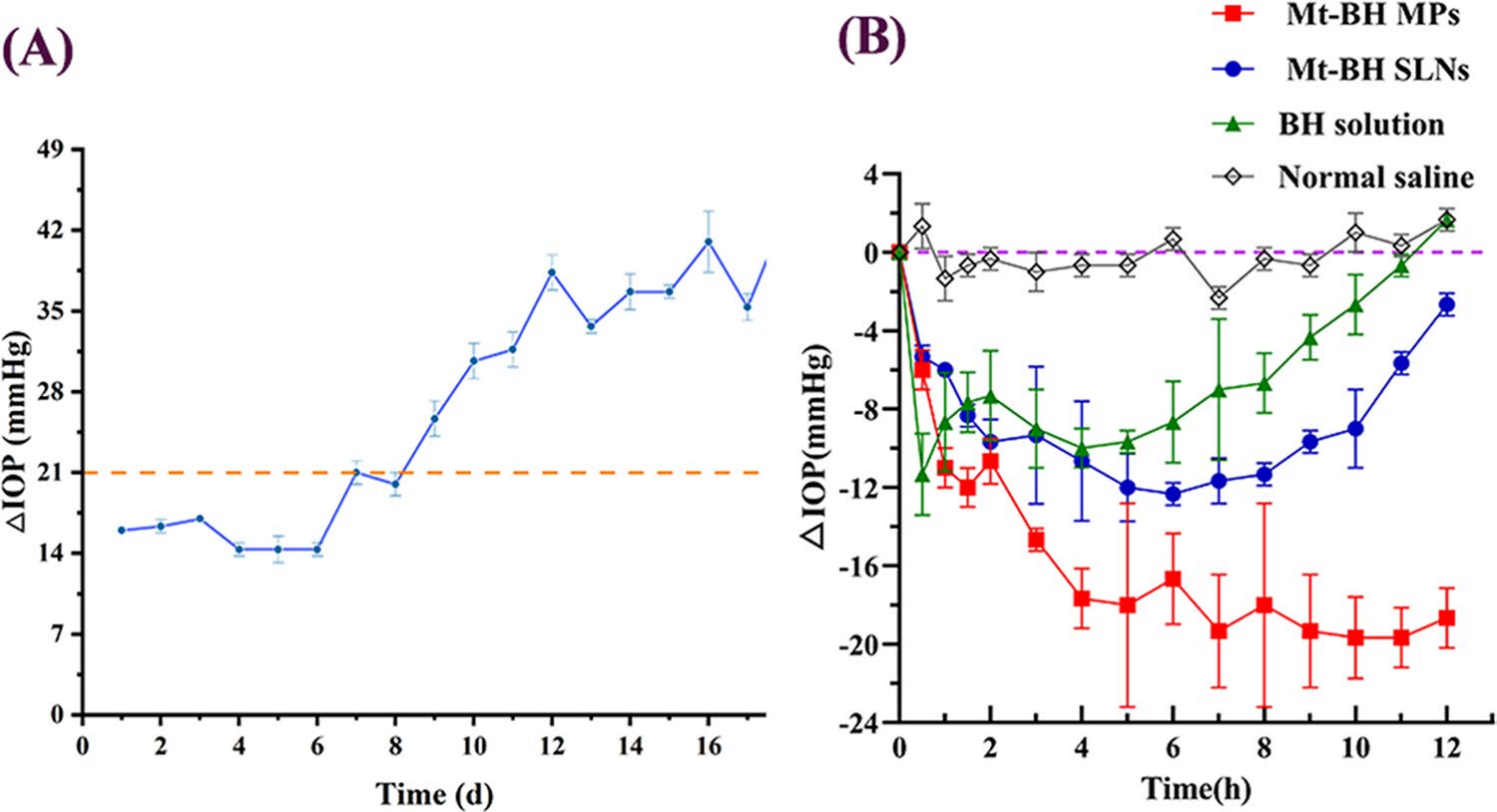
A: Rabbit chronic high intraocular pressure model. B: IOP-lowering effects of BHC solution, MT-BHC MPs and MT-BHC SLNs eye drops after topical administration (mean ± SD, *n* = 3).

Finally, we measured the IOP in New Zealand rabbits after a single dose and analyzed the resulting differences in efficacy. As shown in ***Figure 5***(A), the IOP remained above 22 mmHg for more than 7 days, indicating that the rabbit model of chronic ocular hypertension was successfully established with compound carbomer solution. In Figure 5(B), saline administration (control group) resulted only in a slight fluctuation of IOP within 12 h without any IOP lowering effect. The rapid release of BHC from solution resulted in a quick reduction of IOP by 12 mmHg units at 0.5 h, but gradually reduced the lowering effect after 4 h. Compared to the short-lived IOP lowering effect of the BHC solution, MT-BHC MPs and MT-BHC SLNs resulted in more significant IOP lowering with the IOP lowering effect of MT-BHC MPs being significantly (*p*** **<** 0**.05) greater than that of MT-BHC SLNs eye drops. It is worth mentioning that the MT-BHC MPs group still lowered IOP by around 18 mmHg at 12 h, eventually enabling IOP to reach the normal range, while the IOP in the MT-BHC SLNs group tended to recover after 6 h.

Furthermore, the area under the IOP reduction curve (AUC) of the selected formulations is summarized in ***Table 5***. Compared with BHC solution (78.36 ± 4.214 mmHg·h), AUC of MT-BHC SLNs and MT-BHC MPs was significantly improved, almost 1.4 and 2.5 times of BHC solution, respectively. In addition, the AUC of MT-BHC MPs was significantly 1.8 times compared to that of MT-BHC SLNs (*p* < 0.0001). The result revealed that of these three formulations, MT-BHC MPs have the most consistently sustained IOP-lowering effect with the highest bioavailability.

**Table 5.** Date analysis of the IOP-lowering effects.

	BHC solution	MT-BHC SLNs	MT-BHC MPs
Area under the Curve(AUC) ((mmHg·h) ± SD)	78.36 ± 4.214	106.8 ± 3.422^**^	194.8 ± 6.636^****^^####^

Mean values ± SD, n = 3 per group; ^*^P< 0.05, ^**^ P< 0.01, ^***^ P< 0.001, and ^****^ P< 0.0001 vs. the BHC solution. ^#^ p< 0.05, ^##^ p< 0.01, ^###^ p< 0.001, and ^####^ P<0.0001 vs. the MT-BHC SLNs. .

Although both MT-BHC MPs and MT-BHC SLNs eye drops have the characteristics of dual slow and controlled release, the same drug concentration and almost the same parameters of osmotic pressure and pH value, the IOP reduction effect of the two is different. As discussed earlier, the physicochemical properties of the MT-BHC MPs eye drop themselves play an important role in increasing their precorneal retention capacity. On the one hand, the cumulative release of drug from MT-BHC MPs in vitro was higher than that from MT-BHC SLNs before 3 h, suggesting a faster therapeutic concentration. Subsequent sustained release maintained an effective therapeutic drug dose resulting in a steady IOP reduction without any toxic effects caused by excessive drug. On the other hand, the longer precorneal retention time together with the unique physicochemical properties of the MT-BHC MPs offer the potential for sustained drug release and efficacy. These results were also corroborated by tear drug pharmacokinetics. The MT-BHC MPs resulted in the highest drug content in the tears during the first 1 h which was maintained for the longest time, providing excellent IOP-lowering efficacy. In summary, compared with the BHC solution, both the MT-BHC MPs and MT-BHC SLNs produced better efficacy, and the IOP-lowering effect of the MT-BHC MPs was the most stable and long-lasting.

### Ocular biocompatibility

#### Draize test

The irritation test scores after single and multiple eye drop administration are listed in ***Table 6***. After a single administration, the irritation scores of the three preparation groups were all 0 within 72 h, and after repeated administration (twice per day for 21 days), the average score was less than 3, indicating that BHC solution, MT-BHC MPs and MT-BHC SLNs should be nonirritant when used for long-term glaucoma management.

**Table 6.** Ocular irritation scores in rabbits (single and multiple administration, *n* = 3).

	Single dosing				Multiple dosing			
	Saline	BHC aqueous solution	MT-BHC SLNs	MT-BHC MPs	Saline	BHC aqueous solution	MT-BHC SLNs	MT-BHC MPs
Cornea	0	0	0	0	0	0	0	0
Iris	0	0	0	0	0	0	0	0
Conjunctival congestion	0	0	0	0	0	1.67	0.67	0
Edema	0	0	0	0	0.33	0	0	0
Secretions	0	0	0	0	0	0.33	0.33	0.33
Total	0	0	0	0	0.33	2	1	0.33

#### Histopathology examination

**Figure 6.**
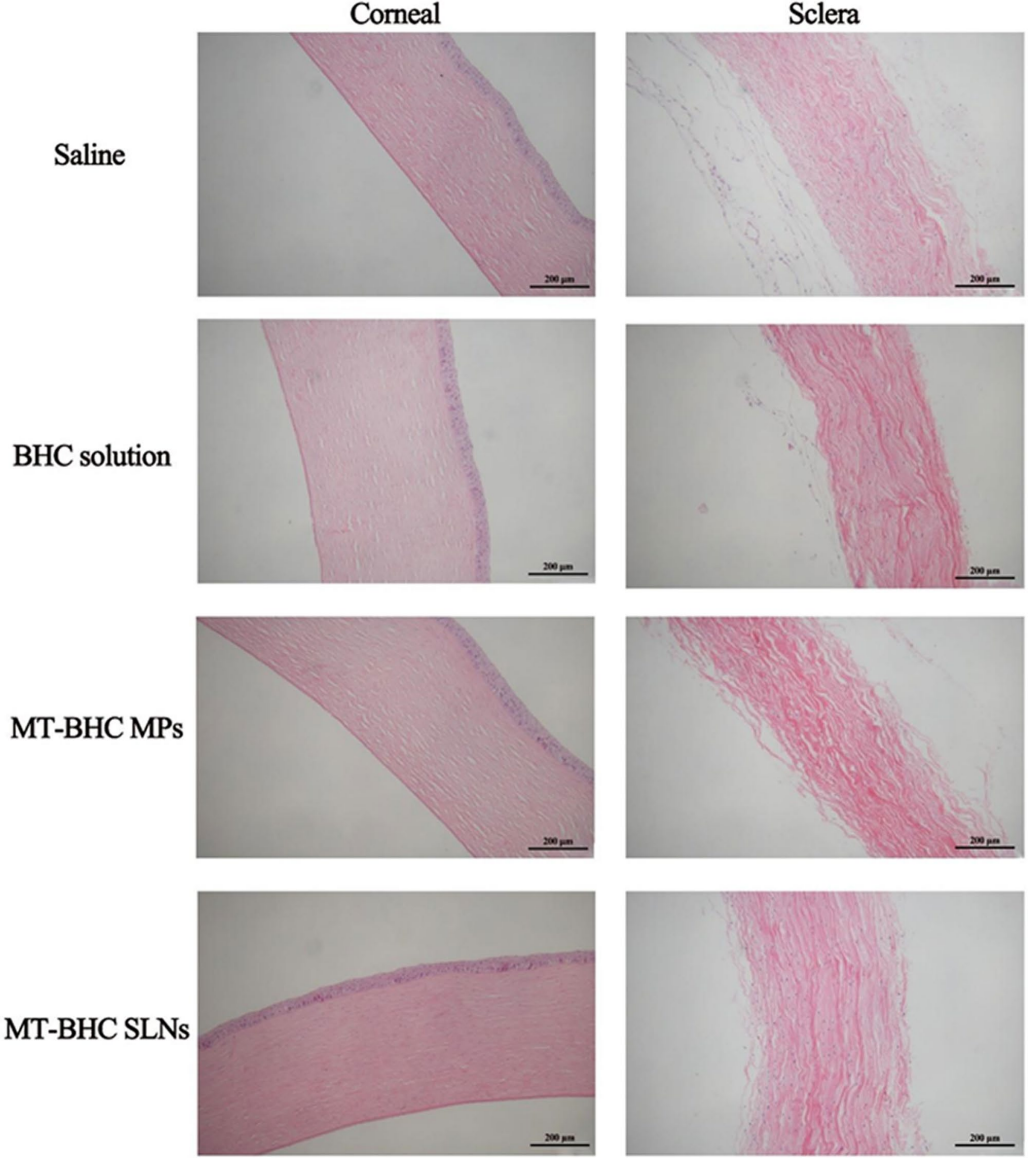
Corneal and scleral tissue sections after repeated eye drop administration.

Corneal and scleral sections after repeated administration are depicted in ***Figure 6***. Corneal epithelial cells and stromal collagen fibers were arranged regularly in all groups without any inflammation. Moreover, collagen fibrils in the scleral tissue sections of all groups were arranged in an orderly manner with no inflammatory cell infiltration. There were no obvious signs of structural damage in either cornea or scleral tissue suggesting good biocompatibility of the formulations.

#### Blink experiment

Results from the blink experiments are shown in ***Table 7***. Within 1 min after administration, the blink frequency in the saline group was 3 ± 2 min^−1^. The blink frequency after application of the BHC solution was slightly higher than for the saline group, but there was no significant difference between them. The blink frequency after application of MT-BHC MPs was slightly increased compared to the MT-BHC SLNs group. The slight increase may be caused by the fact that the size of MT-BHC MPs is larger than that of MT-BHC SLNs. There was no significant difference in blink times between saline, BHC solution, MT-BHC SLNs, and MT-BHC MPs, suggesting that the preparations did not produce any significant foreign body sensation or irritation after administration. In combination with the previously described results of irritation scores and the tissue sections, delivery systems containing the same drug concentrations showed no significant toxicity, indicating that the ocular bio-safety of EUD polymers is comparable to that of lipid carrier materials.

**Table 7.** Blink frequency after eye drop administration (data expressed as the mean values ± SD, *n* = 6).

Samples	Blink frequency (min^–1^)
Saline	3 ± 2
BHC solution	7 ± 2
MT-BHC MPs	7 ± 3
MT-BHC SLNs	6 ± 2

## Conclusion

In this research, MT-BHC MPs and MT-BHC SLNs were prepared by embedding betaxolol hydrochloride into montmorillonite through an ion-exchange mechanism. The in vitro release experiment showed that MT-BHC MPs and MT-BHC SLNs exhibited some burst release before changing to more sustained drug release with the burst from the MT-BHC MPs in the early stage beneficial to quickly achieve the drug required drug concentration to efficiently lower the IOP. Compared with the BHC solution, both positively charged MT-BHC MPs and MT-BHC SLNs significantly extended the precorneal retention time with the disappeared fluorescent signal only after 135 and 45 min respectively, owing to their higher viscosity, lower surface tension, and longer precorneal diffusion time. Tear elimination pharmacokinetics showed that drug could still be detected in the tears 360 min after MT-BHC SLNs administration and 480 min after MT-BHC MPs administration, proving their longer precorneal retention time and long-lasting stability, with MT-BHC MPs being the most superior, due to their strong hydrophobic surface promoting the micro-interaction with the cornea. Based on the micro-interaction between the preparation and the tear film and the cornea, it was proposed that this may shift the tear film flow pattern, namely the drug clearance from hydrostatic layer turnover to mucin layer turnover control. The hydrophobic nature of MT-BHC MPs further facilitated interactions with the highly hydrophobic corneal epithelium, resulting in the release of more therapeutic molecules and ultimately a higher IOP lowering effect. Ocular irritation experiments demonstrated that neither MT-BHC MPs nor MT-BHC SLNs was significant toxic at the same drug concentration, indicating that the ocular biosafety of EUD polymers was comparable to that of lipid carrier materials. Overall, the dual sustained-release MT-BHC MPs may have great potential in glaucoma management.
